# The Influence of Tide, Wind, and Habitat on the Abundance and Foraging Rate of Three Species of Imperiled Plovers in Southwest Florida, USA

**DOI:** 10.3390/ani13223548

**Published:** 2023-11-17

**Authors:** Jayden L. Jech, Elizabeth A. Forys

**Affiliations:** Biology and Environmental Studies Disciplines, Eckerd College, St. Petersburg, FL 33711, USA; jljech@eckerd.edu

**Keywords:** barrier island, foraging, nonbreeding season, Piping Plover, Snowy Plover, Wilson’s Plover

## Abstract

**Simple Summary:**

This research studied three species of imperiled shorebirds—Piping Plover, Snowy Plover, and Wilson’s Plover—that are similar in appearance and share habitats outside the breeding season. Researchers counted the number of these birds on a barrier island in Southwest Florida and examined how tide and wind affected their abundance and foraging behavior. They found that all three species were more likely to be found on the mudflats during lower tides and on the beach during higher tides. All of the feeding observed was on the mudflat, but one of the species, Wilson’s Plovers, was never observed feeding during the study. The high densities of these plover species are likely due to the mudflat habitat that provides more food, a beach habitat that provides a relatively safe location to roost, and the location of the barrier island, which is surrounded by other good places to forage and rest. While each species used the barrier island’s habitats slightly differently, the high densities of all three species present an opportunity to have a large conservation impact by protecting this dynamic area.

**Abstract:**

Piping Plover (*Charadrius melodus*), Snowy Plover (*Charadrius nivosus*), and Wilson’s Plover (*Charadrius wilsonia*) are imperiled species that overlap in both their range and habitat outside the breeding season. The purpose of this research was to document the abundance of these species at a barrier island in Southwest Florida, USA, and to examine the influence of tide and wind on both their abundance and foraging. We walked ~700 m surveys through tidal mudflat and adjacent beach semiweekly at 0730–1030 from 24 September 2021–4 March 2022. During these 38 surveys, Piping Plovers were the most abundant of the three species, and the average number counted was 34 (SD 17.3) compared with 11 (SD 6.3) Snowy Plovers and 14 (SD 14.1) Wilson’s Plovers. All of the species were more likely to be found on the mudflats during low tides and the beach during high tides. Our findings suggest the high densities of Piping, Snowy, and Wilson’s Plover are due to a mudflat habitat that provides high availability of prey, a beach habitat that provides a relatively safe location to roost, and the context of the barrier island, which is surrounded by other suitable habitats. While each species used the barrier island’s habitats slightly differently, the high densities of all three species present an opportunity to have a large conservation impact by protecting this dynamic area.

## 1. Introduction

Piping Plover (*Charadrius melodus*), Snowy Plover (*Charadrius nivosus*), and Wilson’s Plover (*Charadrius wilsonia*) are morphologically similar, imperiled species that overlap in both their range and habitat outside the breeding season [1,2,3]. Wilson’s Plovers have the longest body and culmen (upper bill), followed by Piping Plovers, and Snowy Plovers are the smallest [4,5,6]. Most research into the habitat use of plovers has been completed during the breeding season, but nonbreeding habitat is critical to plovers, as they spend most of their lives in these areas [7,8]. Nonbreeding site selection can be related to food availability, presence of predators, disturbances, and habitat quality [8,9,10].

Piping Plovers nest along the mid to north Atlantic Coast, Great Plains, and Great Lakes and are currently listed as being federally threatened or endangered depending on the population [11]. They are a relatively short-distance migrant that commonly winters along the Atlantic Coast of the United States from North Carolina south, along the entire Gulf Coast, and on several islands in the Caribbean [11,12]. Research into wintering Piping Plovers has indicated they are primarily found in coastal habitats and forage on algal, sand, and mud flats at lower tides [13,14,15].

Snowy Plovers nest along the U.S. Pacific Coast, Gulf Coast, and some inland breeding sites. They are one of the rarest species of shorebirds in North America, and the subspecies that breeds in the Pacific is listed as federally threatened [16]. East Coast Snowy Plovers are listed in nearly every state they occur in [5]. While some do migrate short distances, others stay in the same general area year round [5]. Nonbreeding surveys have found Snowy Plovers occur along the Atlantic coast of Florida, throughout the Gulf Coast of the U.S., and at various sites in the Caribbean [17]. Snowy Plovers use a diversity of habitats, including sandy beaches, sand/salt flats, mudflats, gravel shores, and algal mats.

Wilson’s Plovers breed along the Atlantic Coast from Virginia to the south, throughout the Gulf of Mexico and Caribbean, and in coastal areas in northern South America. During the winter, some of the more northern birds migrate, but the migration patterns of most of the species is unknown [6]. Wilson’s Plovers have been designated as a bird of conservation concern by the U.S. Fish and Wildlife Service [18] and are on several state’s endangered species lists [6]. Their nonbreeding distribution is not known, but they have been recorded throughout the Gulf Coast [3,19].

All three species are visual predators and typically use the “run-stop-peck” or “stop-run-peck” method of foraging [13,20,21]. While not well studied, the diet of Piping Plovers and Snowy Plovers is similar and varies by location and season, but polychaete worms and other invertebrates appear to be common items [5,10,11,22]. Also poorly studied, Wilson’s Plovers also eat invertebrates but prefer larger species, particularly fiddler crabs (*Uca* spp.) [6,23,24].

Identifying how these imperiled species use these overwintering areas is critical for their continued conservation [2,8]. Throughout the Gulf of Mexico, there is also the unique opportunity to find and protect areas where all species occur, maximizing the time and effort needed [1]. Located on the Gulf of Mexico, Southwest Florida, and more specifically, the Tampa Bay region is known to support high numbers of shorebirds despite being very developed [25,26,27,28]. Fort De Soto County Park is a largely undeveloped 460 ha barrier island complex located at the mouth of Tampa Bay. The Fort De Soto area supports high numbers of all three species of plovers outside the breeding season [19].

The purpose of this paper is to (1) document the abundance of these imperiled species during the nonbreeding season in the habitats where they are most likely to occur (tidal flat and open beach), (2) determine the influence of tide and wind on their use of these habitats, and (3) quantify the foraging rate while in these habitats and what role tide and wind play. Based on the morphological and diet similarity between Piping and Snowy Plovers, we hypothesize that these two species will be more similar to each other than to Wilson’s Plovers in terms of their abundance on the tidal flats and beach and their response to tide and wind.

## 2. Materials and Methods

**Figure 1:**
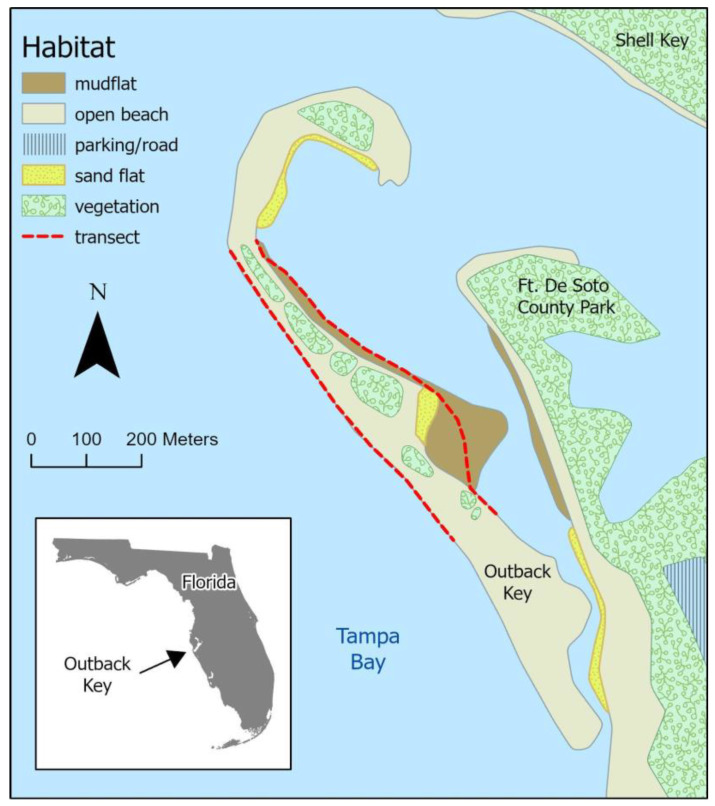
Map of Outback Key, Florida, the major habitat types, and the transect routes.

All research was conducted on Outback Key, a small (100 ha) barrier island just north of Fort De Soto County Park (Figure 1). To its immediate north is Shell Key Preserve, an undeveloped barrier island. This newly accreted low-lying island has a relatively thin beach with exposed sand on the west side that faces the mouth of Tampa Bay and low dunes with emerging vegetation (saltgrass, *Distichlis spicata*; sea oats, *Uniola paniculata*; seashore dropseed, *Sporobolus virginicus*). It also has an extensive tidally influenced flat, the majority of which is covered in fine-grained sediment that is <80% sand and categorized as a mudflat, with a smaller portion that has >95% sand and is categorized as a salt flat [13,14]. We will refer to this tidally influenced habitat as the “mudflat” throughout this paper.

While this area is not an official critical habitat for the Piping Plover [29], data collected during the Florida Winter Bird Survey [19] from 2020–2022 placed Outback Key in the top 15 for all beaches surveyed in Florida for number of Piping, Snowy and Wilson’s Plovers. During the period of time we conducted our surveys, Outback Key was an island with no terrestrial mammalian predators. While it was adjacent to a county park, visitors had to arrive by boat or cross a 7–10 m channel; thus, there were relatively few people on the key during the weekdays, but more people on the weekends when the boat traffic was greater.

We conducted surveys semiweekly of the mudflat flat and adjacent open beach from 0730–1030 from 24 September 2021–4 March 2022, with a 1-month gap in late December/early January. We walked approximately 700 m of mudflat and a similar length of beach (Figure 1) and recorded the total number of individuals of each study species. Binoculars (10 × 42) were used during surveys, and care was taken to avoid areas where birds were present; thus, the transect varied depending on the location of each plover species and other shorebirds. To determine whether species abundance varied similarly among surveys, we conducted paired Pearson’s correlations between each species. To account for the increased likelihood of type I errors arising from doing multiple correlations, we applied Bonferroni correction, and our corrected alpha value was 0.017.

Following the abundance surveys, two focal foraging surveys were completed per species. Focal surveys were conducted by randomly selecting individuals to record foraging behaviors and the number of pecks. Previous research has indicated that the peck rate can be representative of the intake or foraging rate of a plover when the prey of the plover is too small to identify during the survey [30,31]. Focal surveys were three minutes per individual, based on a prior study of foraging behavior, which included a two-minute survey per foraging individual [32]. An additional minute was added to include more foraging data. If individuals of the species were not present after 15 min, no focal survey was conducted. We compared the average peck rates between the species using a nonparametric Wilcoxon test because it was count data.

We evaluated the influence of wind speed (km/h), tide height (m), and the interaction of tide and wind on the number of Piping, Snowy, and Wilson’s Plovers on the mudflat and beach habitat using a generalized linear model (GLM) with the base model of R (4.3.1). We used a Poisson distribution because we had count data. Prior to analysis, we screened for multicollinearity and standardized the tide and wind variables. We then plotted the interaction of tide and wind using the R package ggeffects [33]. To analyze the peck rate data, we ran a generalized linear mixed model (GLMM) with tide and wind as fixed effects and date as a random effect, as we sampled several individuals on the same date. For this analysis, we used the R package lmer [34].

Tide data was sourced from the National Oceanic and Atmospheric Administration (NOAA) tides and current station at Mullet Key, Tampa Bay, Florida—Station ID: 8,726,364 and was estimated at the time prior to the survey at 0700 during the survey day [35]. Wind speed was recorded from The Weather Channel of St. Petersburg, Florida, real-time weather report during the beginning of each survey [36]. During the surveys, we also recorded any color-banded birds using a telephoto lens camera and reported these to the individual who banded the bird on the breeding grounds.

## 3. Results

**Figure 2:**
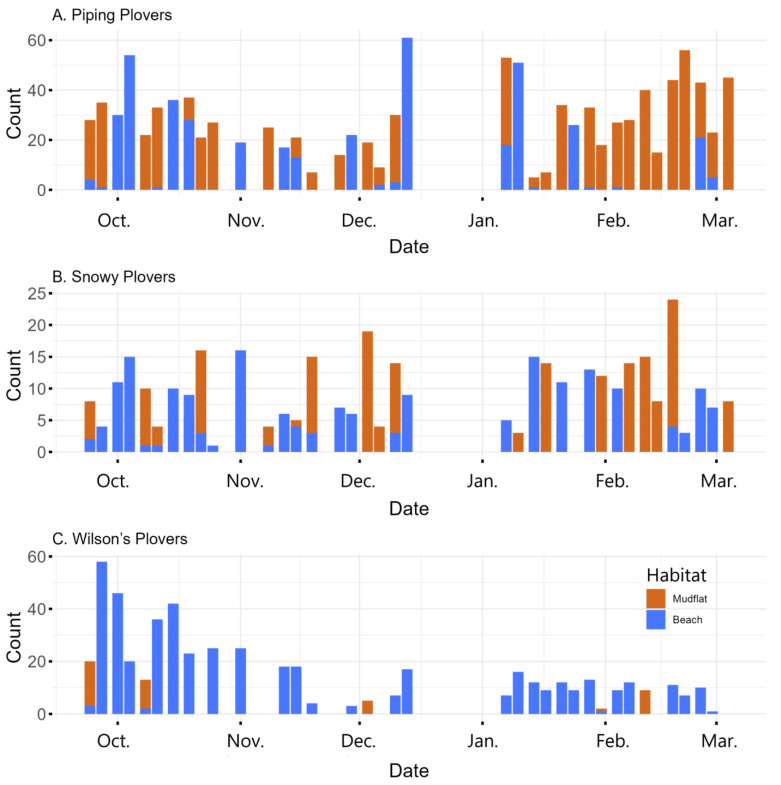
The number of plovers seen during each survey on the mudflat and adjacent beach at Outback Key, Pinellas County, Florida.

We conducted 38 surveys, and each 1400 m survey took ~1 h. During surveys, Piping Plovers were the most abundant of the three species, and the average number counted was 34 (SD 17.3) compared with 11 (SD 6.3) Snowy Plovers and 14 (SD 14.1) Wilson’s Plovers (Figure 2). The numbers of Piping Plovers and Snowy Plovers were similar throughout the nonbreeding season (September–March), while the number of Wilson’s Plovers decreased after October. Twice as many Piping Plovers were seen on the mudflat compared with the beach, Snowy Plovers were nearly equally seen on the mudflat and beach, and only 12% of the Wilson’s Plovers were seen on the mudflat; the rest were seen on the beach (Figure 2). There were no significant correlations between pairs of plovers counted on the mudflat.

For plovers counted on the beach, the number of Piping and Snowy Plovers seen during each survey were significantly correlated (Bonferroni converted alpha = 0.017, Pearson r = 0.391, *p* = 0.015). There were strong, positive correlations between Piping–Wilson’s (Pearson r = 0.355, *p* = 0.029) and Snowy–Wilson’s (Pearson r = 0.357, *p* = 0.028); however, they were not statistically significant.

Thirteen banded Piping Plovers were recorded during the study: eight were from the Great Lakes population, four from the Atlantic Coast, and one from the Northern Great Plains. Most of the banded Piping Plovers were seen throughout the study, but three were only seen once, and these were all from the Great Lakes. The Great Lakes Population is ~2000 km to the north of Outback Key, the Northern Great Plains population is ~2500 km to the northwest, and the Atlantic population is ~2000 km to the northeast.

Twelve of the Snowy Plovers were banded. Seven were local birds who nested at Outback, four were from >300 km away in the Panhandle region of northwest Florida, and one was from Sanibel Island, Florida (170 km to the south). Most stayed for several months, and all of the residents and one of the migrants stayed the entire nonbreeding season. Only two Wilson’s Plovers were banded; one was a resident who stayed all of the nonbreeding season, and one was a migrant from the Panhandle of Florida who was seen only once.

Tide was statistically significant for each GLM that predicted the abundance of Piping, Snowy, and Wilson’s Plovers (*p* < 0.05, Table 1). Because we sampled at the same time during the 38 surveys, we were present for the full range of tides (−0.19 to 0.52 m, 0.16 ± 0.19 m, mean ± SD). The data were normally distributed (Shapiro–Wilk normality test, W = 0.96, *p* = 0.16). Wind was statistically significant for most of the GLMs that predicted the abundance of Piping, Snowy, and Wilson’s Plovers (*p* < 0.05, Table 1). Wind ranged from 0 to 33.8 km/hr (12.07 ± 7.72, mean ± SD). Wind speed was also normally distributed (W = 0.95, *p* = 0.06).

**Figure 3:**
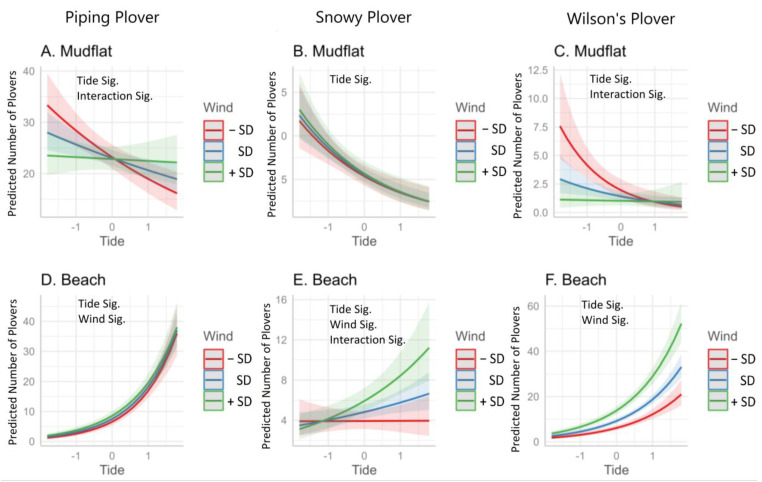
Predicted marginal effects and 95% confidence intervals (shaded areas) of standardized tide and wind on the number of Piping, Snowy, and Wilson’s Plovers. The red line presents the influence of tide when wind is low (1 SD below the mean), the blue is when it is average, and the green is when the wind is high (1 SD above the mean).

Piping and Wilson’s Plovers exhibited similar patterns with respect to their abundance due to tide and wind. Both species significantly increased on the mudflats as the tide decreased, and this increase was greatest when the wind was low. On the beach, they significantly increased when the tide and wind were higher (Figure 3, Table 1).

Snowy Plovers significantly increased on the mudflats as the tide decreased but were not significantly affected by wind speed. On the beach, they significantly increased as wind and tide increased, and the highest number was seen when wind speeds and tide were highest (Figure 3, Table 1).

**Figure 4:**
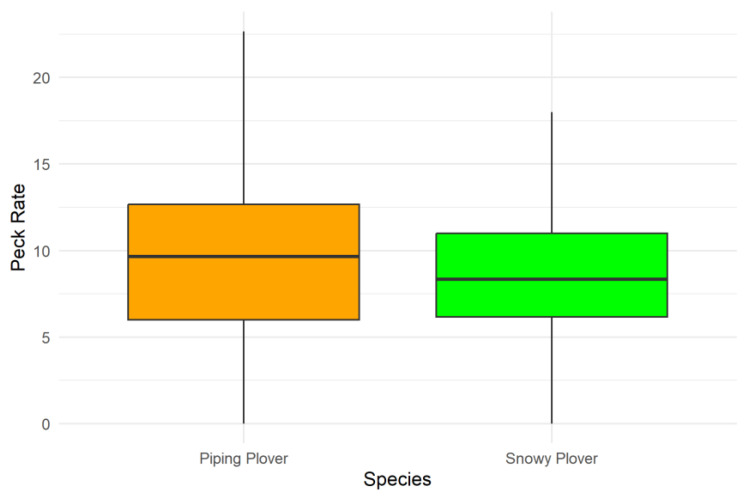
Number of pecks/minute (Piping Plover *n* = 101, Snowy Plover *n* = 59). The median is shown by the horizontal line inside the box. The box shows the interquartile range, and the vertical lines are the range.

Piping and Snowy Plovers were only observed foraging on the mudflats. Wilson’s Plovers were not observed foraging during the study. We did not observe any foraging by any of the species on the beach. Piping Plovers had a median peck rate of 9.7/min (*n* = 101, Figure 4), and Snowy Plovers had a median peck rate of 8.3 pecks/min (*n* = 59, Figure 4). The species did not significantly differ in their peck rates (W = 3458, *p* = 0.09). In addition, tide and wind had no significant effect on the peck rate of either the Piping Plover or the Snowy Plover (Table 2).

## 4. Discussion

The mudflat at Outback Key supported high densities of Piping and Snowy Plovers, and the beach supported high densities of all three species. Piping and Snowy Plovers used the mudflat for foraging and the beach for roosting. Wilson’s Plovers were rarely seen on the mudflat but roosted on the beach. The average number/km of Piping, Snowy, and Wilson’s Plovers was very high in comparison to similar studies on barrier islands within the United States Gulf Coast (Table 3).

While Wilson’s Plovers were more abundant at the start of the nonbreeding season, their numbers decreased to about 15–20 individuals for the rest of the season, indicating some individuals were likely using Outback as a stopover site during the early part of fall migration. In contrast, the number of Piping Plovers and Snowy Plovers was fairly consistent throughout the nonbreeding season, indicating that most of the birds were overwintering on Outback Key (Figure 2). The high number of Piping Plovers in this one small area is consistent with other nonbreeding studies that found that small areas may be of disproportionally large importance to wintering plovers [37]. This was reinforced by the presence of the same banded birds throughout the nonbreeding season.

Banded Piping Plovers were primarily from the Great Lakes, which is the smallest and most endangered population [38]. Previous studies have found that Great Lake breeders tend to overwinter on the Atlantic Coast rather than the Gulf of Mexico [12,39,40,41]. Our sample size is small and covers only one nonbreeding season, but these initial results suggest a multiyear study is warranted. Recent analysis has determined that while there are broad patterns of migration and strong nonbreeding site fidelity, Piping Plovers from different breeding locations do spend portions of the nonbreeding season in similar locations [39]. The banded Snowy Plovers and Wilson’s Plovers at our study site indicated that there was a mix of resident and migratory individuals, which is consistent with the literature [5,6].

Overall, tide had a larger influence on the number of the three species of plovers on the mudflat than wind (Figure 3). For Piping Plover and Wilson’s Plover, the interaction between tide and wind was also significant. The lower the tide, the more birds were on the mudflat, but on windy days, fewer Piping and Wilson’s Plovers were on the mudflat, irrespective of the tide.

Our results are in agreement with other studies of Piping Plovers [13,42,43,44,45]. Research elsewhere has documented that the abundance of birds on the flats increases with lower tides, and wind is also occasionally important [13]. There are no published studies of the influence of tide or wind on Snowy Plover habitat in the nonbreeding season. It is interesting to note that they used the mudflat at low tides, even when the winds were relatively high.

Piping and Snowy Plover foraging increased on the mudflats during lower tides (Figure 3). The foraging peck rate of both species was not influenced by tide or wind, indicating that if Piping and Snowy Plovers were present on the mudflat, they were actively foraging. While the number of pecks/minute was very similar to studies of Piping Plovers, some studies found that peck rates increased during lower tides [42,43].

We found some support for our hypothesis that Piping and Snowy Plovers are more similar to each other than to Wilson’s Plovers, as their numbers were significantly correlated on the beach, and their peck rates on the mudflat were very similar. There were slight differences in how Piping Plovers and Snowy Plovers responded to environmental variables, and this partially explains the finding that their abundance on the mudflat was not significantly correlated [46,47].

Relatively few Wilsons’ Plovers were found on the mudflat, and we did not observe them actively foraging (Figure 2). This is likely due to the patchy nature of their dominant prey item, fiddler crabs, genus *Uca* [23,48,49], and our methodology of only surveying for a few hours in the morning [23]. Studies of Wilson’s Plovers in South America have routinely found they are more likely to forage at night because of the availability of their prey and possibly due to diurnal predators [50], although researchers elsewhere have observed them foraging during the day [49].

On the beach, both wind and tide significantly influenced the number of each species. As the tide and wind increased, Piping, Snowy, and Wilson’s Plover were more likely to seek refuge on the beach. While we did not measure the distance between individuals on the beach, we noted that roosting plovers were often found together in large mixed-flock roosts, sometimes with other shorebirds, primarily *Calidris* spp. Other studies have found that the larger the flock of roosting shorebirds, the lower the probability of an alarm flight [51,52].

One key independent variable we did not measure was disturbance from humans and potential predators. Human disturbance can decrease both abundance and foraging [22,32]. While Outback Key benefits from being adjacent to Ft. De Soto County Park, where dogs are only allowed in specific areas, and there are rangers to help enforce county rules, we are aware that on the weekends, there was more human use and this likely would decrease both abundance and foraging of all three plover species. In addition, after this study was completed, a storm filled in the channel between the park and Outback Key, and this did increase the number of people on the flats and adjacent beach. While there were relatively few predators on the beach, occasional avian predators (Merlin, *Falco columbarius*) could play a role in abundance [8].

## 5. Conclusions

Research has shown that during the nonbreeding season, plovers in areas with higher habitat quality have higher survival [8]. This habitat quality is influenced not only by the type of habitat and availability of prey but also by the magnitude of human disturbance and predator abundance.

Outback Key, located in Southwest Florida, supports one of the highest densities of Piping, Snowy, and Wilson’s Plover throughout their range in the nonbreeding season. For Piping and Snowy Plovers, this high density appears to be related to the extensive mudflat for foraging during low tides and, for all three species, a relatively disturbance-free beach for roosting during high tides. The location of Outback Key, surrounded by other protected barrier islands, provides plovers with different options for foraging and roosting and likely contributes to the high density [13,14].

As predicted by the Piping Plover literature, all three species were more abundant on the mudflats when tides were lower and on the beaches when tides were higher [13,14,15]. Wind decreased the abundance of Piping Plovers and Wilson’s Plovers on the mudflats and increased all species numbers on the beach. Tide and wind should be taken into account when conducting plover surveys, and correction factors employed or surveys should be repeated and averaged.

Due to Outback Key’s role in supporting Piping, Snowy, and Wilson’s Plovers throughout the nonbreeding season, continued protection and management of the barrier island is crucial for the management of these species. Support for the Gulf of Mexico Avian Monitoring Network and management of other locations where these three species overlap is needed to prioritize management efforts throughout the region [52].

Future research into plover species at Outback Key should determine their diet and prey availability through the day and night. It would be good to replicate this research at multiple locations in Southwest Florida. In addition, studies should look at the influence of human and predator disturbance on abundance. Because the number of people increased dramatically on weekends compared with weekdays, a study that compared the number of plovers during similar tides and winds would help determine the magnitude of humans on these imperiled species and aid with management suggestions [53].

## Figures and Tables

**Table 1:** Results of generalized linear models (using Poisson distribution) of the influence of tide, wind, and the interaction between tide and wind on the abundance of Piping Plovers, Snowy Plovers, and Wilson’s Plovers at the mudflat and beach habitats. Significance: *** for *p* < 0.001, ** for *p* < 0.01, and * for *p* < 0.05.

Species, Habitat	Model	Estimate	SE	*z*-Value	*p*-Value
Piping Plover, mudflat	Intercept	0.35	0.15	2.37	0.0180 *
	Tide	−0.40	0.15	−2.72	0.007 **
	Wind	−0.32	0.18	−1.79	0.073
	Interaction	0.35	0.16	2.14	0.032 *
Piping Plover, beach	Intercept	2.01	0.07	29.67	<0.001 ***
	Tide	0.89	0.06	15.42	<0.001 ***
	Wind	0.13	0.06	2.16	0.032 *
	Interaction	−0.06	0.05	−1.22	0.222
Snowy Plover, mudflat	Intercept	1.71	0.07	23.60	<0.001 *
	Tide	−0.45	0.07	−6.13	<0.001 *
	Wind	0.03	0.09	0.34	0.737
	Interaction	−0.01	0.07	−0.18	0.856
Snowy Plover, beach	Intercept	1.57	0.07	21.00	<0.001 ***
	Tide	0.17	0.07	2.40	0.016 *
	Wind	0.20	0.08	2.60	0.009 **
	Interaction	0.18	0.07	2.52	0.012 *
Wilson’s Plover, mudflat	Intercept	3.13	0.03	92.39	<0.001 ***
	Tide	−0.11	0.03	−3.16	0.002 **
	Wind	0.01	0.04	−0.20	0.838
	Interaction	0.09	0.03	2.65	0.008 *
Wilson’s Plover, beach	Intercept	2.21	0.06	36.49	<0.001 ***
	Tide	0.71	0.05	13.00	<0.001 ***
	Wind	0.41	0.05	8.00	<0.001 ***
	Interaction	0.03	0.04	0.62	0.532

**Table 2:** Results of the GLMs that determined the influence of tide, wind, and the interaction of tide and wind on the foraging rate of Piping and Snowy Plovers.

Species, Habitat	Model	Estimate	SE	*z*-Value	*p*-Value
Piping Plover, mudflat	Intercept	3.24	0.84	38.49	<0.001
	Tide	−0.01	0.08	−0.07	0.942
	Wind	0.14	0.08	1.8	0.072
	Interaction	0.02	0.07	0.242	0.802
Snowy Plover, mudflat	Intercept	3.15	0.1	31.46	<0.001
	Tide	−0.17	0.1	−1.71	0.088
	Wind	0.06	0.1	0.55	0.582
	Interaction	0.12	0.06	1.94	0.052

**Table 3:** A comparison of the abundance of plovers from our study and two similar studies.

Location (USA), Length of Transect, Citation	Piping Plover (Average Abundance)	Snowy Plover (Average Abundance)	Wilson’s Plover (Average Abundance)
SW Florida, 1.4 km, this study	34.1	11.0	14.3
Mississippi, 1.6 km, [2]	3.8	2.7	0.6
Texas, 40 km, [3]	9.4	3.9	0.1

## Data Availability

Data are available upon request from the primary author.
